# Chlorhexidine bathing for the prevention of colonization and infection with multidrug-resistant microorganisms in a hematopoietic stem cell transplantation unit over a 9-year period: Impact on chlorhexidine susceptibility

**DOI:** 10.1097/MD.0000000000005271

**Published:** 2016-11-18

**Authors:** Elisa Teixeira Mendes, Otavio T. Ranzani, Ana Paula Marchi, Mariama Tomaz da Silva, José Ulysses Amigo Filho, Tânia Alves, Thais Guimarães, Anna S. Levin, Silvia Figueiredo Costa

**Affiliations:** a Department of Infectious Diseases; b Pulmonary Division, Heart Institute, Hospital das Clínicas, University of São Paulo; c Laboratory of Bacteriology, Department of Infectious Diseases; d HSCT Department, Hospital das Clinicas; e Department of Infectious, Diseases of Hospital das Clinicas, University of São Paulo, São Paulo, Brazil.

**Keywords:** chlorhexidine bath, hospital infection, hematopoietic stem cell transplantation infection, multidrug-resistant bacteria, transplant infection

## Abstract

Health care associated infections (HAIs) are currently among the major challenges to the care of hematopoietic stem cell transplantation (HSCT) patients. The objective of the present study was to evaluate the impact of 2% chlorhexidine (CHG) bathing on the incidence of colonization and infection with vancomycin-resistant *Enterococcus* (VRE), multidrug-resistant (MDR) gram-negative pathogens, and to evaluate their CHG minimum inhibitory concentration (MIC) after the intervention.

A quasi-experimental study with duration of 9 years was conducted. VRE colonization and infection, HAI rates, and MDR gram-negative infection were evaluated by interrupted time series analysis. The antibacterial susceptibility profile and mechanism of resistance to CHG were analyzed in both periods by the agar dilution method in the presence or absence of the efflux pump inhibitor carbonyl cyanide-m-chlorophenyl hydrazone (CCCP) and presence of efflux pumps (qacA/E, qacA, qacE, cepA, AdeA, AdeB, and AdeC) by polymerase chain reaction (PCR).

The VRE colonization and infection rates were significantly reduced in the postintervention period (*P* = 0.001). However, gram-negative MDR rates in the unit increased in the last years of the study. The CHG MICs for VRE increased during the period of exposure to the antiseptic. A higher MIC at baseline period was observed in MDR gram-negative strains. The emergence of a monoclonal *Pseudomonas aeruginosa* clone was observed in the second period.

Concluding, CHG bathing was efficient regarding VRE colonization and infection, whereas no similar results were found with MDR gram-negative bacteria.

## 1 Introduction

Health care associated infections (HAIs) are currently among the major challenges to the quality of hematopoietic stem cell transplantation (HSCT) patient care.^[1]^ Multidrug-resistant (MDR) bacteria, such as vancomycin-resistant *Enterococcus* (VRE),^[2]^ and carbapenem-resistant gram-negative bacteria,^[3]^ constitute the leading etiologic agents of bloodstream infections (BSIs) in some bone marrow transplantation (BMT) health centers.^[4,5]^

Due to these conditions, daily bathing with 2% chlorhexidine (CHG) has been proposed to reduce the colonizing bacterial burden in critically ill patients^[6,7]^ and, thus, reduce the rates of infection and cross-transmission. CHG bath impact has been evaluated, particularly in intensive care units (ICUs).^[8–10]^ Notably, some studies report reduced colonization with MDR microorganisms and a general reduction of bacteremia, mainly due to coagulase-negative staphylococci (CNS).^[8,9]^

To date, clinical prospective studies that evaluate the real-life impact of prolonged CHG use on the development of bacterial resistance to antiseptics are lacking, and studies approaching HSCT population, especially non-ICU patients, remain scarce in the literature.^[9,11,12]^

A major concern about the introduction of CHG bath has been the potential increase in selection pressures. Indeed, cases of CHG minimum inhibitory concentration (MIC) increase have been reported mainly for *Staphylococcus aureus*^[13]^ and *Enterococci* spp.^[14]^ isolates. Efflux-pump genes (*cepA*, *qacAE*, and *qacE*) have been identified and associated with gram-negative bacterial strains with high CHG MICs.^[15,16]^

The objective of the study was to evaluate the impact of CHG bathing on colonization and infection by MDR bacteria in the BMT ward of a university hospital. We also aimed to assess the CHG MICs for MDR bacteria and presence of efflux pump genes, both before and after the implementation of daily bathing with chlorhexidine.

## 2 Materials and methods

### 2.1 Study design

This is a quasi-experimental intervention study comparing a 4.5-year pre-intervention period (January 2005–July 2009), during which inpatients of the BMT ward were bathed with regular liquid soap, with an equally long intervention period (August 2009–December 2013), during which inpatients of this ward were routinely bathed with 2% chlorhexidine digluconate solution.

### 2.2 Setting

This study was conducted in the University of São Paulo Medical Centre (Hospital das Clínicas da Faculdade de Medicina da Universidade de São Paulo—HC-FMUSP) with 2200 beds, of which 1000 are located in the Central Institute, where the study was conducted. BMT unit is a ward of adult patients with occasional pediatric transplants until 2007. It performs 10 to 12 transplants a month, both autologous and allogeneic. This study was approved by the hospital's Ethics Committee for Research Projects (CAPPesq—Comissão de Ética e Análise de Projetos de Pesquisa, São Paulo, Brasil number: 08362413.9.0000.0068).

### 2.3 Study population

Patients of both periods were compared as to gender, age, underlying hematological diseases, types of transplants, comorbidities, and 30-day and 1-year post-HSCT mortality rates.

### 2.4 Procedures

The chlorhexidine bathing procedure (with 2% chlorhexidine digluconate) was standardized by the nursing team of the HSCT ward and the Hospital Infection Control Department during August 2009 and maintained during the entire intervention period. Following orientation of the nursing team at admission, patients performed their own chlorhexidine bath daily. In our hospital, 2% CHG is also used in the antisepsis of invasive procedures such as central venous catheters insertion, surgery, and biopsies.

### 2.5 Outcomes

Primary outcome was the incidence density (ID) of VRE colonization and infection. Secondary outcome was MDR gram-negative bacteria infection, including *Acinetobacter baumannii*, *Pseudomonas aeruginosa*, and *Klebsiella pneumoniae*, resistant to carbapenems at any site.

Further endpoints included the ID of recorded HAIs by any given microorganism at any site, chlorhexidine MICs of isolates, and the characterization of mechanisms of resistance of bacteria isolated before and after the intervention.

### 2.6 Definitions

Monthly IDs were calculated according using the exemplary equation: ID = number of cases/1000 patient-days.

The definitions for HAIs were those used by the Centres for Disease Control and Prevention (CDC).^[17]^

In the BMT unit, only VRE colonization was weekly monitored with anal swabs from all inpatients in the study period. The swabs were inoculated in a 6 μg/mL vancomycin supplemented broth.^[18]^

All patients diagnosed as colonized or infected with MDR microorganisms were kept under cohort contact precautions until hospital discharge. International standard definitions for acquired resistance was used to MDR determination.^[19]^ We did not to include MRSA (methicillin-resistant *Staphylococcus aureus*) in our analysis, by its low incidence in the unit only 2 infections during the study period (<2.5%).^[4]^

### 2.7 Laboratory analysis of chlorhexidine MICs

MICs for CHG were determined for 127 isolates (46 *Pseudomonas aeruginosa*; 48 VRE; 27 *Klebsiella pneumoniae*; and 6 *Acinetobacter baumannii* n: 6). Postintervention strains were compared with control strains, represented by bacteria isolated during the pre-intervention period from the unit.

The samples evaluated were those that were available at the research laboratory in the pre- and postintervention (microbiology laboratory/Institute of Tropical Medicine University of São Paulo).

There were no *K. pneumoniae* and *A baumanni* isolated in pre-intervention period at the unit, neither in surveillance or clinical cultures. These MDR gram-negatives were introduced in our BMT unit after 2010.

The MICs were determined by the agar dilution test by incorporating serial logarithmic concentrations of chlorhexidine in Mueller-Hinton agar (MHA) culture medium, which was then distributed onto individual Petri dishes as in Abuzaid et al.^[15]^ The tested concentrations ranged from 0 to 256 mg/L, and controls, *K pneumoniae* ATCC13883 (chlorhexidine MIC of 16 μg/mL), and *Escherichia coli* ATCC25922 (chlorhexidine MIC of 2 μg/mL) were included in each experiment.

Each experiment was performed in triplicate and discarded if the ATCC strains varied by more than 1 dilution.

### 2.8 Evaluation of chlorhexidine MICs in the presence of the efflux pump inhibitor carbonyl cyanide-m-chlorophenyl hydrazone (CCCP) in the culture medium

The influence of the efflux pump on the antibacterial susceptibility profile of the studied strains to chlorhexidine was evaluated by assessing the MICs of chlorhexidine in the presence of the efflux pump inhibitor CCCP (Sigma-Aldrich, Saint Louis, MO, USA) in the culture medium.

Specifically, CCCP was diluted with 1 mL of distilled water and added to the MHA plates at a final concentration of 10 mg/L.^[15]^ These plates were prepared with chlorhexidine concentrations ranging from 0 to 256 mg/mL. The influence of an efflux pump on the CHG MIC for a given bacterial strain was defined as an MIC reduction of at least 4-fold in the presence of CCCP.^[15]^

### 2.9 Evaluation of the mechanisms of resistance to chlorhexidine

The mechanisms of resistance were evaluated using polymerase chain reaction (PCR). The primers are listed in Table 1.^[20–23]^

**Table 1:**
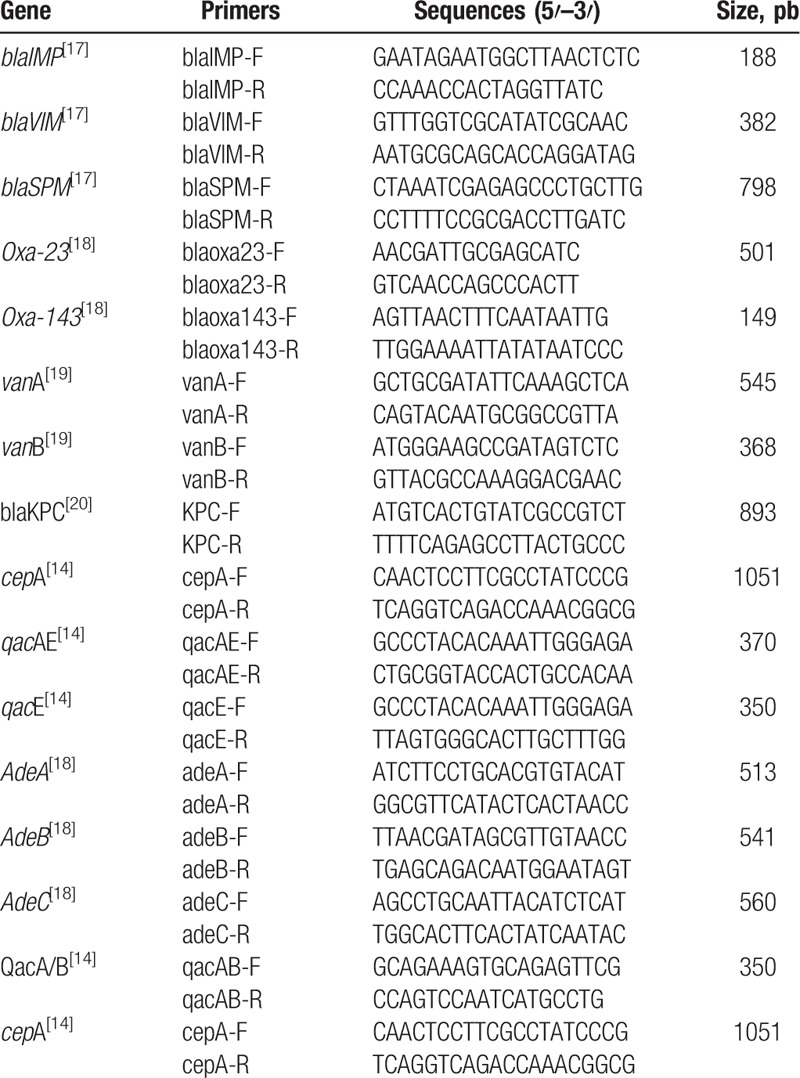
Primers used to evaluate genes that encode resistance to antibiotics and that encode efflux pumps associated with chlorhexidine resistance.

### 2.10 Evaluation of clonality

The clonality of samples was characterized by pulsed-field gel electrophoresis (PFGE). The PFGE patterns were analyzed with Bionumerics version 7.1 (Applied-Maths, Sint-Martens-Latem, Belgium). DNA fragments were manually curated and normalized using the molecular weight standard from each gel. Restrictions enzymes and parameters used were SMA-I for VRE, SPE-I for *P aeruginosa*, XBA-I for *K pneumoniae*, and Apa-I for *A baumannii*.^[24–28]^A 1.5% band tolerance and 0.5% optimization were used. Cluster analysis was performed by the unweighted pair group method using arithmetic averages (UPGMA). Isolates were considered to be genetically related if the Dice coefficient was ≥ 80%.^[24–27]^

### 2.11 Statistical analysis

In the descriptive analysis, categorical and continuous data were presented as percentages and mean ± SD values [or medians and interquartile ranges (IQRs)], respectively. The pre- and postintervention periods were compared. The categorical variables were compared using a Chi-square test or Fisher exact test, as appropriate. The quantitative continuous variables were compared using an unpaired *t* test and the Mann–Whitney *U* test for normally and non-normally distributed variables, respectively.

The association between the implementation of chlorhexidine bathing and the endpoints, considering time-related changes, such as general improvements in patient care and secular trends, was assessed by interrupted time series analysis (ITS).^[28,29]^ The data were aggregated at equal time intervals (months) and we used the autoregressive integrated moving average (ARIMA) model. Therefore, we could evaluate the secular trend (coefficient Beta-1), the immediate change after intervention started (coefficient Beta-2), and the long-term effect of the intervention (coefficient Beta-3).

We checked the assumptions required for ARIMA models using Phillips–Perron, the Kwiatkowski–Phillips–Schmidt–Shin (KPSS), and Augmented Dickey–Fuller tests.^[29]^ The autocorrelation was checked by visual inspection of autocorrelograms and partial autocorrelograms of the series and its residuals. The White neural network test was used to test for neglected nonlinearity. We also checked for seasonal or cyclical effects by decomposing our series. The Ljung–Box Q test was run to evaluate a lack of fit of the final ARIMA.^[29]^ Because of the outbreak of *P aeruginosa* during the intervention period, we pre-specified to use a nonlinear time series for gram-negative bacteria. Therefore, we used a general additive model (GAM), allowing for autocorrelation.^[28]^

The database was constructed, processed, and organized with Microsoft-Excel 2010. Statistical and time series analyses were performed using Epi.Info 3.5.3 (Atlanta, GA, USA), SPSS 21.0 package for Windows (Armonk, New York, USA), and the R project (Vienna, Austria) for statistical computing.

## 3 Results

### 3.1 General Characteristics of the Studied Population

During the 9 years of the study (4.5 years pre- and 4.5 years postintervention), 1393 HSCTs were performed in the unit, with 870 patients in the pre-intervention period and 523 patients in the intervention period. The clinical and epidemiological characteristics of the patients are listed in Table 2. Despite the higher total number of patients during pre-intervention period, we had a greater number of patient-days in the intervention period (15,600 vs 10,195 patient-days). This may mean a longer hospital stay and more severe and complex cases such as allogeneic HSCT in the intervention period.

**Table 2:**
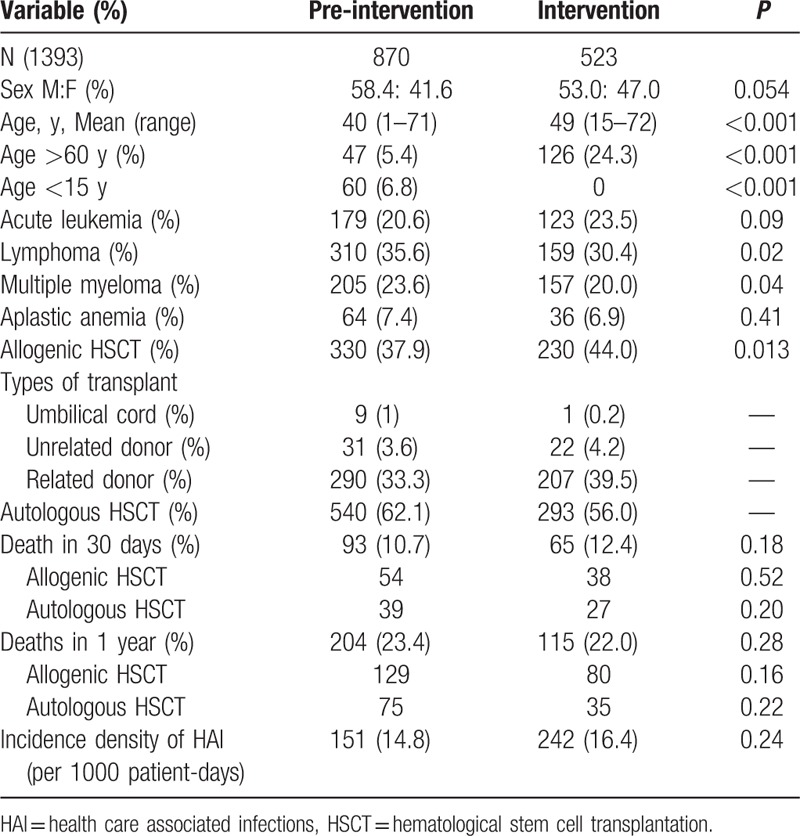
Epidemiological and clinical characteristics of 1393 patients submitted to HSCT in Hospital das Clínicas, University of São Paulo, Brazil (2005–2013) to evaluate a daily bathing with chlorhexidine as an intervention to reduce colonization and infection by antimicrobial-resistant microorganisms.

Notwithstanding this difference, the 30-day and 1-year mortality rates did not differ between the studied periods, and the overall HAI rate was also not different, suggesting that patients of both groups exhibited similar long-term profiles of severity and prognosis, irrespective of the type of transplantation performed.

**Figure 1:**
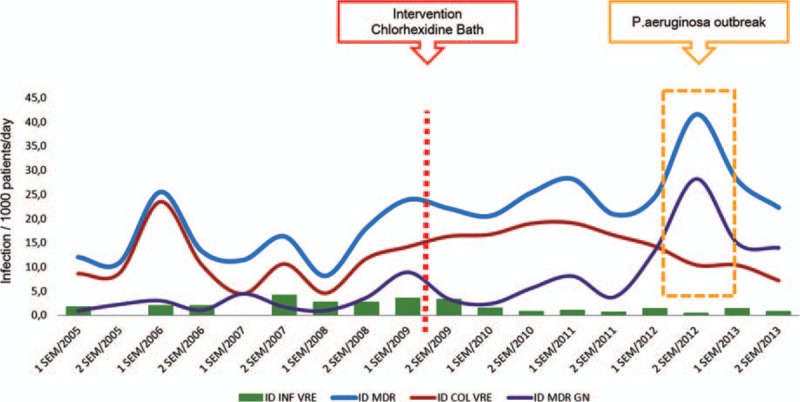
Bianual incidence density of MDR infection and colonization in pre- and postintervention periods, in BMT unit, HC-FMUSP, 2005–2013. Col = colonization, GN = gram-negative bacteria, ID = Incidence density, Inf = Infection, MDR = Multidrug-resistant bacteria, VRE = Vancomycin-resistent *Enterococcus*.

The biannual distribution of infection rates of MDR microorganisms is shown in Fig. 1. The increased infection rates of MDR gram-negative bacteria are mainly due to an outbreak of *P aeruginosa* BSIs that occurred from December 2011 to January 2013. During this outbreak, 29 cases of *P aeruginosa* bacteraemia were recorded, especially within the first days after HSCT,^[30]^ with 65% mortality.

### 3.2 Time series

**Figure 2:**
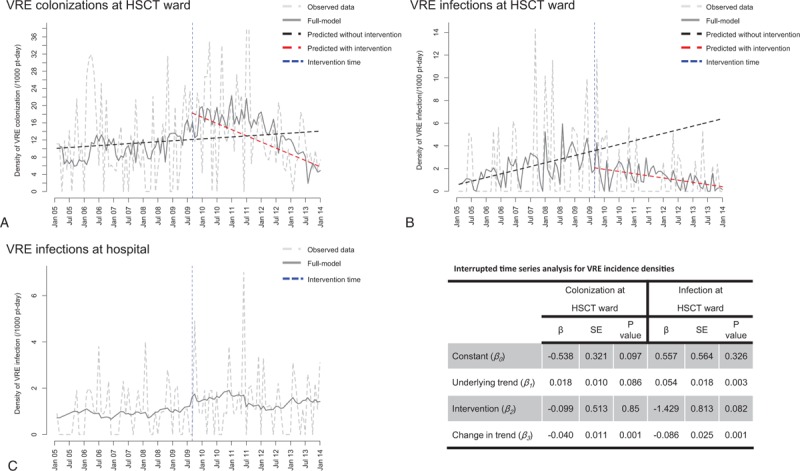
Time series of VRE colonization (A), VRE infection (B) in pre- and postintervention periods, in bone marrow transplant ward, and global hospital VRE incidence (C) in HC-FMUSP, 2005–2013.

Figure 2A and B show the temporal distribution of VRE colonization and infection rates in the BMT unit. There was a significant decrease in the incidence of colonization (Change in trend: Beta-3 = −0.040, *P* *=* 0.001) and infection (Change in trend: Beta-3 = −0.086, *P* *=* 0.001) during the intervention period. This reduction did not occur in the global hospital rates (Fig. 2C).

In contrast, the infection rates of MDR gram-negative bacteria increased in the last two years of the study in the BMT unit. Indeed, we observed a non-linear underlying trend for MDR gram-negative infections. Even after excluding the *P aeruginosa* outbreak, the postintervention period exhibited the highest rates (*P* < 0.001) of MDR gram-negative bacteria. The BSI ID in the HSCT unit was similar in both periods (*P* = 0.31).

### 3.3 Evaluation of the antibacterial susceptibility profile and mechanisms of resistance to chlorhexidine

Table 3 describes chlorhexidine MICs in the pre- and intervention periods, both in the presence and in the absence of the efflux pump inhibitor CCCP. Antimicrobial resistance genes were *SPM* in 30% of the tested *P aeruginosa*, *OXA 23* in 83% of *A baumannii*, *KPC* in 100% of *K pneumonia*, and *Van A* in 100% of *Enterococcus faecium* tested. VRE isolates exhibited a considerably lower baseline chlorhexidine MIC than gram-negative bacteria, and their MIC50 increased by 2 dilutions in the intervention period. The chlorhexidine MIC reduction with CCCP was significantly higher in intervention period for VRE isolates (90% response). MIC50 and MIC90 for *P aeruginosa* were identical in the pre- and intervention periods (32 and 64 μg/mL, respectively).

**Table 3:**
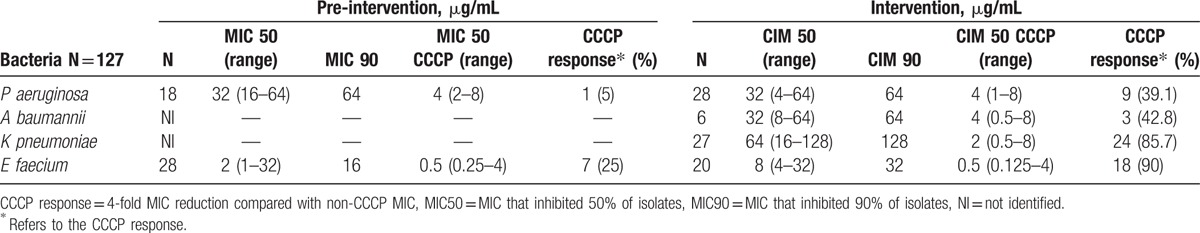
Minimal inhibitory concentrations (MIC) of chlorhexidine and effect of the efflux pump inhibitor CCCP on MIC of bacteria isolated in the pre-intervention and intervention periods in a Bone Marrow Transplant unit, Hospital das Clínicas, University of São Paulo, Brazil (2005–2013).

### 3.4 Mechanisms of resistance to chlorhexidine

We observed a positive correlation between the presence of *cep*A and the response to CCCP. Specifically, of the 25 *cep*A-positive strains, 68% exhibited a reduction of their MIC value by 4 dilutions in the presence of CCCP (Table 4).

**Table 4:**
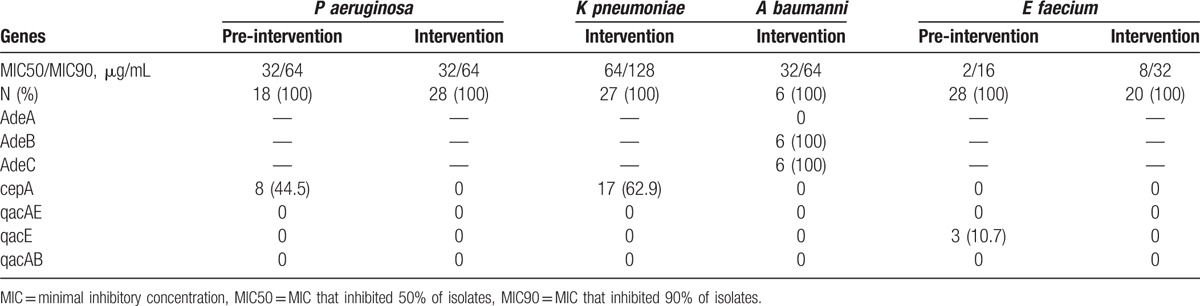
Frequency of chlorhexidine resistance genes found in bacteria isolated in the pre-intervention and intervention periods, Bone Marrow Transplant unit, Hospital das Clínicas, University of São Paulo, Brazil (2005–2013).

CepA was found in 44.5% of *P aeruginosas* tested in the pre-intervention and in none tested in intervention period. CepA was also detected in 62.9% and 42.4% of *K pneumoniae* and *A aumannii*, respectively. The efflux pumps AdeB and C were detected in all *A baumannii* strains in intervention period and only one half of them tested in pre-intervention period. AdeA was only found in the pre-intervention group (51%).

The QacE efflux pump was observed in a small proportion of pre-intervention VRE isolates (10%) and its association with the MICs could not be established. QacAB and QacAE were not detected.

**Figure 3:**
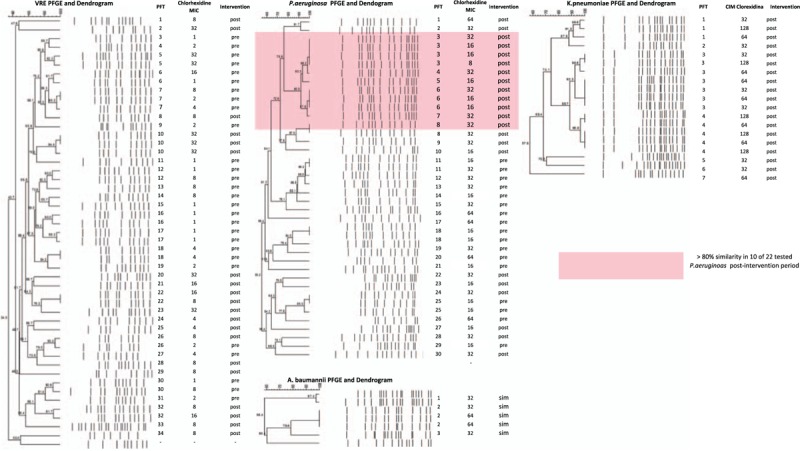
(A–D) PFGE and Dendrogram of VRE, *P aeruginosa*, *K pneumonia*, and *A baumannii*, respectively.

Regarding clonality, the VRE, *K pneumoniae,* and *A baumannii* isolates were polyclonal. With respect to the dendrogram of *P aeruginosa* strains (Fig. 3B), a cluster with >80% similarity was seen in 10 of the 22 evaluated isolates in the intervention period.

## 4 Discussion

Our study shows that daily chlorhexidine bathing led to a significant reduction in rates of colonization and infection by vancomycin-resistant enterococci (VRE) in a bone marrow transplant unit. We consider this an important finding for HSCT population, as, in some centers, VRE is the main bacteraemia-causing agent,^[2]^ especially due to important vancomycin use and recurrent gastrointestinal tract injury, such as graft versus host disease (GVHD) and mucositis.^[1]^

CHG bathing has previously been shown to reduce the incidence of VRE in ICUs.^[31,32]^ However, data on the use of CHG on HSCT patients are scarce and controversial.^[9,11,12]^ A prospective multicenter study evaluating BMT inpatient unit found no impact with the use of CHG bathing.^[9]^ In contrast, a quasi-experimental study showed a nonsignificant reduction of VRE rates.^[11]^ In both cases, the observation periods lasted 6 months or less.

Our study is one of the few that evaluated the use of CHG bathing outside the ICU setting, using shower bath and liquid soap formula. Few studies have evaluated this method in the literature.^[33]^

Similar to the observations by other authors,^[8–10]^ we found no substantial effect of CHG bathing on the incidence of infection and colonization with MDR gram-negative bacteria.

In our BMT unit, even if we exclude data on the outbreak of carbapenem-resistant *P aeruginosa*,^[30]^ we observed an increase in infections caused by MDR enterobacteria and the appearance of carbapenemase-producing *Klebsiella pneumoniae* (KPC) in the intervention period. These MDR enterobacteria had never occurred in the hospital before 2010. The increase on gram-negative infections was observed in the entire hospital; however, the reduction of VRE incidence in BMT unit in the intervention period may have helped in this epidemiological change.

During both periods, isolates were polyclonal, except for the outbreak *P aeruginosa* clone in the intervention period. It is possible that the extensive use of the antiseptic in the unit may have contributed to the shift from a polyclonal *P aeruginosa* pattern seen in the pre-intervention period to a clonal pattern. This clone presented was virulent, as described in another study.^[34]^ The success of MDR *P aeruginosa* clones has been associated with phenotypic and genetic factors.^[35,36]^

Chlorhexidine is a topical antiseptic that changes the bacteria osmotic equilibrium, reduces their metabolic capacity, and breaks through the bacterial cell membrane, and the presence of efflux pumps is the most important mechanism of resistance.^[6]^ Resistance to antiseptics, in general, is poorly studied, and there are no clearly defined susceptibility break points. Thus, the increase in MICs following exposure to chlorhexidine does not necessarily imply resistance.

The evaluation of the long-term ecological impact of bathing with chlorhexidine is crucial because it is widely used in many formulations in the hospital environment. The increase in the MIC50 by 2 dilutions for VRE in the postintervention period suggests that prolonged exposure to the antiseptic might increase the MIC for these bacteria. This phenomenon has previously been described and can either imply resistance or merely indicate adaptive tolerance mechanisms that may be reversed after the interruption of exposure.^[37]^

In our study, gram-negative bacteria exhibited higher MIC values than gram-positive microorganisms. Studies have previously described this “intrinsic resistance” of gram-negative bacteria^[37]^ not only to CHG but also to other antiseptics. A possible explanation is that the outer membrane of gram-negative bacteria functions as a natural barrier against the entrance of chemical substances, including antiseptics and antimicrobials.^[6]^ This may explain why chlorhexidine bathing was not efficient in reducing the incidence of infection with MDR gram-negative bacteria in most studies.

A significant response to CCCP in our postintervention strains suggest that efflux pump is an important resistance mechanism; however, we did not find many of the most commonly described pumps. It is possible that other efflux-pumps were responsible for this result.

We observed that the *cep*A efflux pump was associated with a response to CCCP in *K pneumoniae* isolates. This finding is consistent with the study by Abuzaid et al^[15]^ who found reduced chlorhexidine MIC values for virtually all strains that carried the *cep*A efflux pump.

To our knowledge, our study is the first to evaluate the impact of an intervention over a long period of time, both with respect to measuring the incidence of HAI and the environmental impact of the antiseptic. All other prospective multicenter studies evaluated interventions over 4 or 6 months, a period that we considered insufficient to evaluate the impact on the microbiota of the units.^[8–10]^ We believe that an evaluation is mainly relevant for very vulnerable units, such as those with HSCT patients.

Our study has limitations. In addition to the fact that the study was performed in only 1 center, the pre-intervention period was retrospective and it was not possible to collected important data such as chlorhexidine and antibiotics consumption. Also, the long observation period might contribute confounding factors to the analysis. In the 9-year period, outbreaks, changes in the microbiota in the hospital, and changes in hospitalization policies in the unit may have occurred, thus changing the epidemiological and patient characteristics in the ward. The increase in MDR gram-negative rates in the intervention period may have influenced the reduction in VRE incidence. However, the MDR gram-negative bacteria increased epidemically throughout the hospital and VRE rates remained stable in other units, only decreased in BMT ward.

In conclusion, chlorhexidine bathing reduced the incidence of VRE in the BMT unit, and is a promising method for the prevention of one of the most incident and difficult to control pathogens in this patient population. This effect did not occur for MDR gram-negative bacteria that became more prevalent in the unit and in the hospital.
